# Early-life inflammatory markers and subsequent psychotic and depressive episodes between 10 to 28 years of age

**DOI:** 10.1016/j.bbih.2022.100528

**Published:** 2022-10-10

**Authors:** Amelia J. Edmondson-Stait, Xueyi Shen, Mark J. Adams, Miruna C. Barbu, Hannah J. Jones, Veronique E. Miron, Judith Allardyce, James P. Boardman, Stephen M. Lawrie, Andrew M. McIntosh, Golam M. Khandaker, Alex S.F. Kwong, Heather C. Whalley

**Affiliations:** a Translational Neuroscience PhD Programme, Centre for Clinical Brain Sciences, University of Edinburgh, UK; b Centre for Clinical Brain Sciences, University of Edinburgh, UK; c National Institute for Health Research Bristol Biomedical Research Centre, At University Hospitals Bristol and Weston NHS Foundation Trust and the University of Bristol, UK; d MRC Integrative Epidemiology Unit, Population Health Sciences, Bristol Medical School, University of Bristol, UK; e Centre for Academic Mental Health, Population Health Sciences, Bristol Medical School, University of Bristol, UK; f Medical Research Council Centre for Reproductive Health, The Queen's Medical Research Institute, The University of Edinburgh, UK

**Keywords:** Inflammation, Depression, Psychotic experiences, ALSPAC, Life course, Omics

## Abstract

Inflammation is implicated in depression and psychosis, including association of childhood inflammatory markers on the subsequent risk of developing symptoms. However, it is unknown whether early-life inflammatory markers are associated with the number of depressive and psychotic symptoms from childhood to adulthood. Using the prospective Avon Longitudinal Study of Children and Parents birth cohort (N = up-to 6401), we have examined longitudinal associations of early-life inflammation [exposures: interleukin-6 (IL-6), C-reactive protein (CRP) levels at age 9y; IL-6 and CRP DNA-methylation (DNAm) scores at birth and age 7y; and IL-6 and CRP polygenic risk scores (PRSs)] with the number of depressive episodes and psychotic experiences (PEs) between ages 10–28 years. Psychiatric outcomes were assessed using the Short Mood and Feelings Questionnaire and Psychotic Like Symptoms Questionnaires, respectively. Exposure-outcome associations were tested using negative binomial models, which were adjusted for metabolic and sociodemographic factors. Serum IL-6 levels at age 9y were associated with the total number of depressive episodes between 10 and 28y in the base model (n = 4835; β = 0.066; 95%CI:0.020–0.113; pFDR = 0.041) which was weaker when adjusting for metabolic and sociodemographic factors. Weak associations were observed between inflammatory markers (serum IL-6 and CRP DNAm scores) and total number of PEs. Other inflammatory markers were not associated with depression or PEs. Early-life inflammatory markers are associated with the burden of depressive episodes and of PEs subsequently from childhood to adulthood. These findings support a potential role of early-life inflammation in the aetiology of depression and psychosis and highlight inflammation as a potential target for treatment and prevention.

## Highlights

• Longitudinal evidence of early-life inflammation with illness burden later in life.
• We explored serum, genetic and epigenetic biomarkers of inflammation.
• Outcomes were observed at 16 time-points (10–28 years of age).
• Serum IL-6 (age 9) strongly associated with total number of depressive episodes.
• CRP DNAm scores weakly associated with total number of psychotic experiences.

## 1 Introduction

Markers for peripheral low-grade chronic inflammation include levels of cytokines, such as IL-6, and acute phase proteins, such as C-reactive protein (CRP), measured in blood serum. These inflammatory markers have consistently shown to associate with many psychiatric disorders, including major depressive disorder and schizophrenia (Yuan et al., 2019). However, the role of these inflammatory markers in the aetiology of these disorders remains inconclusive. It is hypothesised that inflammation may be on causal pathways to depression and psychosis. Mendelian randomisation (MR) studies, that assess causality using genetic instruments, have indeed provided evidence for inflammation being on the causal pathway to psychiatric disorders, rather than the reverse (Perry et al., 2021a). Longitudinal studies have also demonstrated that higher levels of serum IL-6 and CRP in childhood are associated with increased risk of psychotic disorders and depression in early-adulthood, consistent with MR studies (Khandaker et al., 2014, 2018; Metcalf et al., 2017; Perry et al., 2021a, 2021b).

Multiple depressive episodes associate with a more severe depressive phenotype and treatment resistance (Humer et al., 2020; Kendler et al., 2001) and persistent PEs associate with developing severe mental health problems(Kalman et al., 2019). Therefore, it is important to understand potential biological mechanisms that may increase the number of depressive episodes or PEs an individual experiences. Additionally, previous studies have shown associations between inflammation and subsequent persistent depressive symptoms or treatment resistance (Haroon et al., 2018; Iob et al., 2020). We extend these studies by investigating the associations between inflammatory markers measured from birth to age 9 years, here on referred to as early-life inflammatory markers, and the total number of subsequent depressive episodes and PEs, measured during an extensive follow-up period.

Additionally, we use multiple ways of indexing inflammation. Previous studies have typically assessed only serum measures of CRP and IL-6 to investigate the associations between inflammation and psychiatric disorders (Khandaker et al., 2014, 2018). However, these serum measures can fluctuate and are affected by factors such as BMI, recent infections, medication and other inflammatory conditions (Sproston and Ashworth, 2018; Visser, 1999). Genetic and epigenetic predictors of immune proteins have been shown to be more robust for assessment of such factors and provide a more stable/long-term proxy for the proteins they predict, compared to serum measures (Kappelmann et al., 2021b; Stevenson et al., 2021). DNA methylation (DNAm) scores and polygenic risk scores (PRSs) can be used as indicators of an individual's epigenetic and genetic risk respectively to a trait or phenotype, or in this case protein level. We use multiple measures of CRP and IL-6, by not only assessing protein levels in serum, but also investigating DNAm scores and PRSs of CRP and IL-6 from multiple early-life time points, to robustly assess the effect of these proteins.

In this study we aimed to determine early-life inflammatory markers associate with the total number of depressive episodes and PEs observed from ages 10–28 years. We utilised a longitudinal cohort, Avon Longitudinal Study of Children and Parents (ALSPAC), with inflammatory markers from birth to age 9 years and prospective data on depressive episodes and PEs measured at 16 time points throughout adolescence into early adulthood (ages 10–28 years). We hypothesised that both acute (serum) and stable/long-term (DNAm scores and PRSs) inflammatory markers will associate with multiple depressive episodes and PEs.

## 2 Materials and methods

### 2.1 Study sample

Pregnant women resident in Avon, UK with expected dates of delivery April 1, 1991 to December 31, 1992 were invited to take part in The Avon Longitudinal Study of Children and Parents (ALSPAC) (Boyd et al., 2013; Fraser et al., 2013; Northstone et al., 2019). The total sample size for analyses using any data collected after the age of seven is therefore 15,454 pregnancies, resulting in 15,589 foetuses. Of these 14,901 were alive at 1 year of age. Further details are described in Supplementary Methods. Demographics of sample individuals used within the current study are shown in Table 1, Table 2, Table 3.

**Table 1:** **Demographics of participants with serum CRP data.** Includes participants with missing data.

Variable	Female	Male	Missing
	N = 2479	N = 2530	N = 10
**Maternal Education**			
CSE/O-level/Vocational	1169 (47%)	1243 (49%)	0 (0%)
A-level/Degree	1012 (41%)	1026 (41%)	0 (0%)
Missing	298 (12%)	261 (10%)	10 (100%)
**BMI (age 9 years)**	17.80 (2.91)	17.36 (2.60)	18.11 (2.05)
Missing	35	19	0
**CRP (age 9 years)**	0.66 (1.08)	0.46 (0.95)	0.60 (0.89)
**Total Depressive Episodes**			
0	1171 (47%)	1727 (68%)	5 (50%)
1	504 (20%)	404 (16%)	1 (10%)
2	283 (11%)	158 (6.2%)	2 (20%)
3	178 (7.2%)	71 (2.8%)	0 (0%)
4	125 (5.0%)	30 (1.2%)	1 (10%)
5	77 (3.1%)	20 (0.8%)	0 (0%)
6	42 (1.7%)	9 (0.4%)	0 (0%)
7	21 (0.8%)	<5 (0.2%)	0 (0%)
8	11 (0.4%)	<5 (<0.1%)	0 (0%)
9	8 (0.3%)	<5 (0%)	0 (0%)
10	<5 (<0.1%)	<5 (0%)	0 (0%)
Missing	58 (2.3%)	106 (4.2%)	1 (10%)
**Total PEs**			
0	1989 (80%)	2082 (82%)	8 (80%)
1	284 (11%)	202 (8.0%)	1 (10%)
2	77 (3.1%)	47 (1.9%)	0 (0%)
3	26 (1.0%)	9 (0.4%)	0 (0%)
4	7 (0.3%)	<5 (0.2%)	0 (0%)
5	9 (0.4%)	<5 (0%)	0 (0%)
6	<5 (0.1%)	<5 (<0.1%)	0 (0%)
7	<5 (<0.1%)	<5 (0%)	0 (0%)
Missing	82 (3.3%)	185 (7.3%)	1 (10%)
*n (%); Mean (SD)*

**Table 2:** **Demographics of participants with serum IL-6 data.** Includes participants with missing data.

Variable	Female	Male	Missing
	N = 2477	N = 2522	N = 10
**Maternal Education**			
CSE/O-level/Vocational	1167 (47%)	1239 (49%)	0 (0%)
A-level/Degree	1012 (41%)	1022 (41%)	0 (0%)
Missing	298 (12%)	261 (10%)	10 (100%)
**BMI (age 9 years)**	17.80 (2.91)	17.36 (2.60)	18.11 (2.05)
Missing	35	19	0
**IL-6 (age 9 years)**	1.35 (1.50)	1.11 (1.39)	1.51 (0.95)
**Total Depressive Episodes**			
0	1169 (47%)	1720 (68%)	5 (50%)
1	504 (20%)	403 (16%)	1 (10%)
2	283 (11%)	158 (6.3%)	2 (20%)
3	178 (7.2%)	71 (2.8%)	0 (0%)
4	125 (5.0%)	30 (1.2%)	1 (10%)
5	77 (3.1%)	20 (0.8%)	0 (0%)
6	42 (1.7%)	9 (0.4%)	0 (0%)
7	21 (0.8%)	<5 (0.2%)	0 (0%)
8	11 (0.4%)	<5 (<0.1%)	0 (0%)
9	8 (0.3%)	<5 (0%)	0 (0%)
10	<5 (<0.1%)	<5 (0%)	0 (0%)
Missing	58 (2.3%)	106 (4.2%)	1 (10%)
**Total PEs**			
0	1987 (80%)	2076 (82%)	8 (80%)
1	284 (11%)	200 (7.9%)	1 (10%)
2	77 (3.1%)	47 (1.9%)	0 (0%)
3	26 (1.0%)	9 (0.4%)	0 (0%)
4	7 (0.3%)	<5 (0.2%)	0 (0%)
5	9 (0.4%)	<5 (0%)	0 (0%)
6	<5 (0.1%)	<5 (<0.1%)	0 (0%)
7	<5 (<0.1%)	<5 (0%)	0 (0%)
Missing	82 (3.3%)	185 (7.3%)	1 (10%)
*n (%); Mean (SD)*

**Table 3:** **Demographics of participants with inflammatory DNAm score data.** Includes number of participants with missing data for each variable. DNAm scores are reported as Z-scores.

Variable	Female	Male
	N = 441	N = 436
**Maternal Education**		
CSE/O-level/Vocational	211 (48%)	199 (46%)
A-level/Degree	203 (46%)	222 (51%)
Missing	27 (6.1%)	15 (3.4%)
**BMI (age 7 years)**	16.30 (2.34)	16.17 (1.84)
Missing	<5	<5
**CRP DNAm score (at birth)**	0.13 (1.00)	−0.13 (0.98)
**CRP DNAm score (age 7 years)**	0.11 (0.95)	−0.11 (1.04)
**IL-6 DNAm score (at birth)**	0.10 (1.01)	−0.10 (0.98)
**IL-6 DNAm score (age 7 years)**	0.00 (1.02)	0.00 (0.98)
**Total Depressive Episodes**		
0	182 (41%)	251 (58%)
1	92 (21%)	89 (20%)
2	62 (14%)	49 (11%)
3	48 (11%)	17 (3.9%)
4	22 (5.0%)	13 (3.0%)
5	13 (2.9%)	10 (2.3%)
6	14 (3.2%)	<5 (0.2%)
7	<5 (0.7%)	<5 (0.2%)
8	<5 (0.5%)	<5 (0%)
10	<5 (0.2%)	<5 (0%)
Missing	<5 (0.5%)	5 (1.1%)
**Total PEs**		
0	356 (81%)	360 (83%)
1	57 (13%)	59 (14%)
2	15 (3.4%)	12 (2.8%)
3	<5 (0.5%)	<5 (0.2%)
4	<5 (0.9%)	<5 (0%)
5	<5 (0.9%)	<5 (0%)
6	<5 (0.5%)	<5 (0%)
Missing	<5 (0.2%)	<5 (0.9%)
*n (%); Mean (SD)*		

### 2.2 Psychiatric outcomes

#### 2.2.1 Measures of depressive episodes

The Short Mood and Feelings Questionnaire (SMFQ) was used to assess self-reported depressive symptoms at 11 time points between the ages of 10–28 years (Supplementary Figs. 1–2, Supplementary Table 1). The SMFQ was administered via the mail or in clinics. There were three clinic time points (ages 10, 12 and 14 years) and eight remote self-reported (mail) time points (ages 17, 18, 19, 22, 23, 24, 26 and 28 years). The SMFQ measures depressive symptoms experienced in the past 2 weeks and comprises of 13 questions. Each question response is a from 0 to 2, where the total summed score ranges from 0 to 26. A depressive episode is defined as a score ≥ 11, as this cut off has previously been shown to have good specificity for predicting depression (Kwong et al., 2019; Turner et al., 2014). Total depressive episodes were calculated by summing the occurrence of a depressive episode at each time point.

#### 2.2.2 Measures of PEs

The Psychotic Like Symptoms Questionnaire (PLIKS-Q) was used to assess psychotic like experiences at 9 time points between the ages of 13–26 years of age (Supplementary Figs. 1–2, Supplementary Table 2). PLIKS-Q was administered via the mail or in clinics. There were three clinic time points (ages 12, 18 and 24 years) and six remote self-reported (mail) time points (ages 11, 13, 14, 16, 21 and 26 years). PLIKS-Q asks about the presence, frequency and context of experiences associated with psychosis. A PE is defined as answering “Yes - Definitely” to questions asking if the participant had heard anything others had not or seen anything others had not, or if the participant had answered “Yes - Definitely” to questions asking if they felt they were be spied upon or followed and that this occurred at least once a month. This has previously been used as a measure of PEs in ALSPAC (Thapar et al., 2012). Total number of PEs were then calculated by summing the occurrence of a PE at each time point.

Sensitivity analyses were conducted to test whether using an interviewer rated definition of PEs, available at the three clinic time points, gave consistent results to the primary analyses. Using additional PLIKS-Q questions asked during the clinic assessments, interviewers rated PE symptoms as either not present, suspected or definitely present. From this a binary variable was derived: definite PE or no/suspected PE. Total number of interviewer rated PEs were calculated for the three clinic time points and regression analysis was then conducted as in the main analysis.

Sensitivity analyses were conducted to test whether an interviewer rated definition of PEs, available at the three clinic time points, gave consistent results to the primary analyses. Using additional PLIKS-Q questions asked during the clinic assessments and these were used to define an alternative definition of PEs determined by the interviewer (binary variable: definite PE or no/suspected PLE). Using this interviewer rated definition for PEs. Regression analysis was then conducted as in the main analysis.

### 2.3 Inflammatory exposures

#### 2.3.1 Serum IL-6 and CRP

Blood samples were collected from individuals at age 9 years (N = 5079; mean age: 9.86 years; SD: 0.31). High sensitivity serum CRP and serum IL-6 reflect acute inflammation and were measured as described previously (Khandaker et al., 2014). CRP and IL-6 were found to be the most commonly increased inflammatory markers in a meta-analysis of inflammatory serum markers across psychiatric disorders (Yuan et al., 2019). Individuals with serum CRP ≥ 10 mg/L (N = 60) were excluded from the main analysis. This is to minimise confounding by chronic inflammatory condition or acute infection (Giollabhui et al., 2020), consistent with previous studies (Khandaker et al., 2014; Perry et al., 2021b). Serum CRP and IL-6 were log transformed to achieve a normal distribution of residuals.

#### 2.3.2 IL-6 and CRP DNA methylation

DNA methylation was quantified in a subsample of individuals (N = 998) from blood samples obtained from cord blood at birth and at age 7 years (mean age: 7.45; SD: 0.13), as described previously (Barker et al., 2018; Relton et al., 2015). DNA methylation scores were calculated by multiplying the DNA methylation M-value with the effect size for each CpG on a phenotype (obtained through independent association analyses), and then summed. Effect sizes for 7 and 35 CpGs, previously shown to associate with CRP and IL-6 respectively in independent samples, were used to calculate the respective DNAm scores (Ligthart et al., 2016; Stevenson et al., 2021). The CRP DNAm score has previously been used and validated in ALSPAC (Barker et al., 2018). There was no overlap in participant samples used to estimate the effect sizes used to generate the CRP and IL-6 DNAm scores with ALSPAC. White blood cell (WBC) type proportion estimates (B-cells, CD4 T-cells, CD8 T-cells, granulocytes, monocytes and natural killer cells) were estimated from DNA methylation data using the Houseman method (Houseman et al., 2012). This uses a prior reference data to estimate WBC proportions in whole blood samples. DNAm principal components (PCs) were calculated by first residualizing standardised DNA methylation M-values (after removing 44,171 cross-reactive or polymorphic probes(Chen et al., 2013) on age, sex and array, and then applying principal component analysis (PCA) on these residuals.

#### 2.3.3 IL-6 and CRP PRSs

Publicly available genome-wide association study (GWAS) summary statistics were downloaded to calculate PRSs. GWASs on circulating levels of CRP and IL-6 in the blood were respectively obtained from UK Biobank (N = 343,524) (downloaded from the Neale lab repository – http://www.nealelab.is/uk-biobank/) and Finish cohorts (total N = 8233) (The Cardiovascular Risk in Young Finns Study and FINRISK) (Ahola-Olli et al., 2017). Where available, GWASs underwent quality control by removing SNPs with minor allele frequency (MAF) < 0.01 and INFO (imputation quality) < 0.8. INFO was not supplied for IL-6 GWAS summary statistics. There was no sample overlap between either of the GWASs used and the ALSPAC cohort.

Genotyping information (including quality control procedures) of ALSPAC has been described in detail elsewhere and is detailed in the Supplementary Methods (Kwong et al., 2021). PRSs were calculated using SbayesR (Lloyd-Jones et al., 2019) on unrelated participants (N = 7975). SBayesR is a Bayesian method that adjusts the beta values in the GWAS based on LD scores from a reference panel. Shrunk sparse LD matrices based on 1.1 million common SNPs in a random sample of 50K unrelated European individuals were downloaded from the GCTB website (https://cnsgenomics.com/software/gctb/) and used as the reference panel. Default values were used for the variables “pi”, “gamma”, “chain-length”, “burn-in” and “out-freq”, (see code available on GitHub). Additional arguments were parsed to the function including “ambiguous-snp” (removes SNPs with ambiguous nucleotides, ie. A/T or G/C), “imputing N" (imputes per-SNP sample size) and “exclude-MHC” (excludes SNPs in the major histocompatibility complex (MHC regions) - Chr6:28–34 Mb). The number of SNPs used to calculate PRSs for CRP and IL-6 were 286,512 and 1,129,461 respectively.

### 2.4 Statistical analysis

**Fig. 1:**
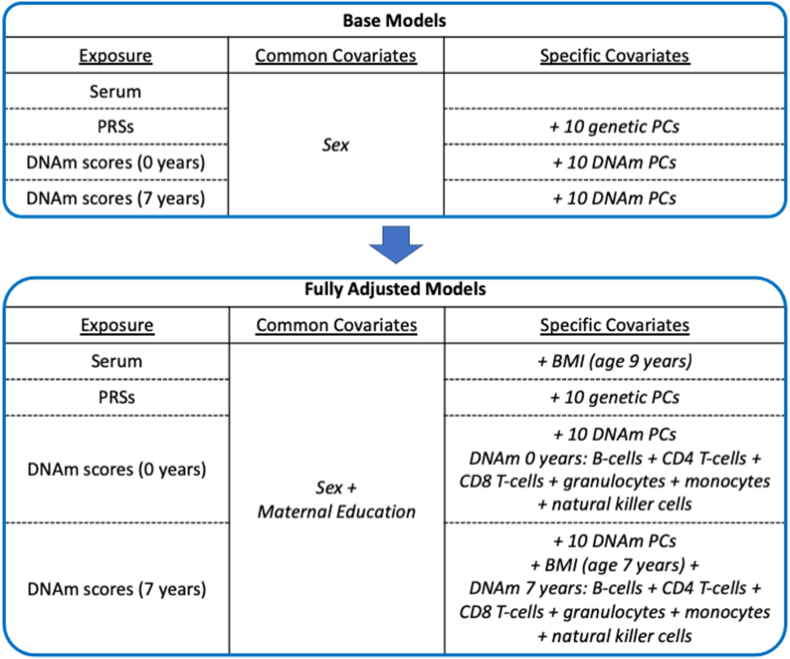
**Covariates used in base and fully adjusted models.** Common and specific covariates used for each different exposure in base and fully adjusted models.

All continuous variables were standardised using z-score scaling to obtain standardised effect sizes (β). Negative binomial models were used to test the associations between inflammatory serum markers (age 9 years), DNAm scores (age 0 and 7 years) and PRSs with subsequent total number of depressive episodes (age 10–28 years) and PEs (age 13–26 years). Two main models were used, the first was a base model covarying for sex only and secondly a fully adjusted model which included BMI (for serum and DNAm scores at age 9 and 7 years respectively) and maternal education, a marker of socioeconomic status (Muscatell et al., 2020; Osimo et al., 2020) (Fig. 1). For the DNAm analysis the base and fully adjusted models also included methodological covariates of 10 DNAm informed PCs and the fully adjusted model additionally included methodological covariates of DNAm WBC estimates. For the PRS analysis the base and fully adjusted models also included 10 genetically informed PCs to adjust for population stratification. Sex, maternal education and BMI were used as covariates as these have all been previously shown to be associated with inflammation or psychiatric symptoms (Beydoun et al., 2020; Osimo et al., 2020). Maternal education was coded as a binary variable as either “CSE/O-level/Vocational education” or “A-level/degree level of education”. Sex was coded as a binary variable as either “Male” or “Female”. BMI (age 7 and 9 years) was calculated by dividing weight (kg) by squared height (meters). Genetic principal components were calculated using PLINK (Purcell et al., 2007).

P-values were corrected for multiple testing using the false discovery rate (FDR) method and significance was deemed FDR corrected p-value (p_FDR_) < 0.05.95% CIs are reported throughout.

Code used for analysis is openly available at https://github.com/AmeliaES/ALSPAC_inflam_2022.

### 2.5 Sensitivity analyses

Pearson's correlation coefficients were calculated to test for correlations between serum CRP and IL-6 with DNAm scores and PRSs of CRP and IL-6 (this is detailed in the Supplementary Information). Additionally, to test whether DNAm scores changed with age Pearson's correlation coefficients were calculated between DNAm scores derived at birth or age 7 years (this is detailed in the Supplementary Information).

Other sensitivity analyses included sex stratification analysis, as sex has been shown to be an important factor associated with inflammation in the context of psychiatric outcomes (Beydoun et al., 2020). In these models, sex was no longer included as a covariate. Sensitivity analyses were also conducted to investigate the effect of potential infection on inflammatory serum markers. In addition to removing individuals with CRP ≥ 10 mg/L, individuals that had self-reported an infection at the time of blood collection or in the preceding week (N = 489) were also excluded. Out of this sample of individuals with reported infections 32 were already excluded in the main analysis due to having CRP ≥ 10 mg/L, therefore only a further 457 individuals were excluded in this sensitivity analysis. Finally, individuals with serum CRP ≥ 10 mg/L (n = 60) that were removed from the main analysis were included as an additional sensitivity test to ascertain if these individuals influenced associations (Giollabhui et al., 2020).

### 2.6 Imputation of missing outcome and covariate data

To address potential bias from sample attrition additional analysis was run using Multiple Imputation Chained Equations (MICE) (van Buuren and Groothuis-Oudshoorn, 2011) to impute missing data for outcomes and covariates (this is detailed in Supplementary Methods and Supplementary Table 3).

## 3 Results

### 3.1 Sample characteristics

In the subsample of individuals with serum data (CRP: N = 5019; IL-6: N = 5009) 18% and 24% of individuals had experienced either one depressive episode or more than one depressive episode respectively (Table 1, Table 2). 9% of individuals had experienced either one PE or more than one PE (Table 1, Table 2). In the subsample of individuals with DNAm data (N = 5019) 21% and 30% of individuals had experienced either one depressive episode or more than one depressive episode respectively (Table 3). 13% and 5% of individuals had experienced either one PE or more than one PE respectively (Table 3). In the subsample of individuals with PRS data (N = 5019) 15% and 36% of individuals had experienced either one depressive episode or more than one depressive episode respectively (Table 4). 8% and 21% of individuals had experienced either one PE or more than one PE respectively (Table 3).

**Table 4:** **Demographics of participants with inflammatory PRSs data**. Includes number of participants with missing data for each variable. PRSs are reported as Z-scores.

Variable	Female	Male	Missing
	N = 3825	N = 4022	N = 15
**Maternal Education**			
CSE/O-level/Vocational	1854 (48%)	2002 (50%)	0 (0%)
A-level/Degree	1433 (37%)	1449 (36%)	0 (0%)
Missing	538 (14%)	571 (14%)	15 (100%)
**CRP PRS**	0.0000 (0.0000)	0.0000 (0.0000)	0.0000 (0.0000)
**IL-6 PRS**	0.0000 (0.0000)	0.0000 (0.0000)	0.0000 (0.0000)
**Total Depressive Episodes**			
0	1610 (42%)	2189 (54%)	6 (40%)
1	683 (18%)	534 (13%)	1 (6.7%)
2	345 (9.0%)	184 (4.6%)	3 (20%)
3	252 (6.6%)	97 (2.4%)	0 (0%)
4	165 (4.3%)	43 (1.1%)	1 (6.7%)
5	98 (2.6%)	32 (0.8%)	0 (0%)
6	59 (1.5%)	11 (0.3%)	0 (0%)
7	32 (0.8%)	6 (0.1%)	0 (0%)
8	20 (0.5%)	<5 (0%)	0 (0%)
9	8 (0.2%)	<5 (<0.1%)	0 (0%)
10	<5 (<0.1%)	<5 (0%)	0 (0%)
Missing	550 (14%)	925 (23%)	4 (27%)
**Total PEs**			
0	2738 (72%)	2738 (68%)	10 (67%)
1	395 (10%)	270 (6.7%)	1 (6.7%)
2	109 (2.8%)	64 (1.6%)	0 (0%)
3	37 (1.0%)	16 (0.4%)	0 (0%)
4	8 (0.2%)	<5 (<0.1%)	0 (0%)
5	12 (0.3%)	<5 (<0.1%)	0 (0%)
6	5 (0.1%)	<5 (<0.1%)	0 (0%)
7	<5 (<0.1%)	<5 (0%)	0 (0%)
Missing	519 (14%)	927 (23%)	4 (27%)
*n (%); Mean (SD)*			

### 3.2 Associations between inflammatory markers and total depressive episodes and PEs

**Fig. 2:**
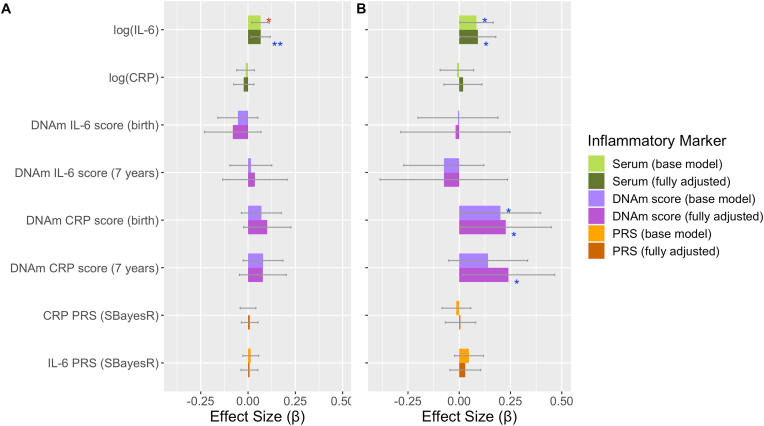
**Association of inflammatory markers with total number of A) depressive episodes and B) PEs.** Standardised effect sizes from negative binomial models with 95% CIs displayed as bars. Red asterisks indicate significance using FDR corrected p-values, blue asterisks indicate significance using uncorrected p-values. Levels of significance: *: p < 0.05; **: p < 0.01. (For interpretation of the references to colour in this figure legend, the reader is referred to the Web version of this article.)

There was strong evidence that serum IL-6 associated with the total number of depressive episodes (ages 10–28 years) in the base model and weaker evidence in the fully adjusted model (p_uncorrected_ < 0.05) (Table 5; Fig. 2A; Supplementary Table 4). We found little evidence for associations between other inflammatory markers (serum CRP, DNAm scores and PRSs) and total depressive episodes (Table 5; Fig. 2A; Supplementary Table 4).

**Table 5:** Summary results for main associations between inflammatory markers and total number of depressive episodes and PEs.

Exposure	Outcome	Covariates	β	95% CI	P (uncorrected)	P (FDR corrected)	Sample Size
log(serum IL-6) (9 years)	Depression episodes	Base Model	0.066	0.020–0.113	0.005	0.041	4835
log(serum IL-6) (9 years)	Depression episodes	Fully Adjusted	0.067	0.016–0.118	0.010	0.076	4264
log(serum IL-6) (9 years)	PEs	Base Model	0.084	0.003–0.166	0.039	0.167	4732
log(serum IL-6) (9 years)	PEs	Fully Adjusted	0.091	0.003–0.178	0.039	0.117	4183
DNAm CRP score (birth)	PEs	Base Model	0.201	0.007–0.397	0.042	0.167	872
DNAm CRP score (birth)	PEs	Fully Adjusted	0.226	0.008–0.449	0.044	0.117	831
DNAm CRP score (7 years)	PEs	Base Model	0.140	−0.051–0.334	0.153	0.374	872
DNAm CRP score (7 years)	PEs	Fully Adjusted	0.240	0.018–0.465	0.036	0.117	828

There was some evidence of association between inflammatory markers and total number of PEs (ages 13–26 years) (Table 5; Fig. 2B; Supplementary Table 4). We observed weak associations (p_uncorrected_ < 0.05) for serum IL-6 and CRP DNAm scores with total number of PEs (Table 5; Fig. 2B; Supplementary Table 4). These associations were observed in both the base and fully adjusted models for all but CRP DNAm scores (7 years) (Table 5; Fig. 2B; Supplementary Table 4). Similar effect sizes were observed for these associations in a sensitivity analysis using an alternative definition of PEs (interviewer rated using additional PLIKS-Q questions) available at three clinic assessments (ages 12, 18 and 24 years) (Supplementary Fig. 3; Supplementary Table 5). We found little evidence of association between the other inflammatory markers and total number of PEs (Fig. 2B; Supplementary Table 4).

**Fig. 3:**
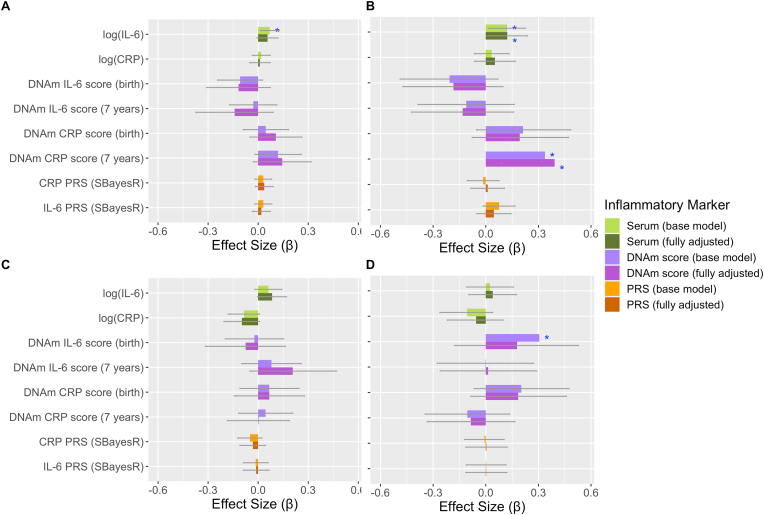
**Association of inflammatory markers with total number of depressive episodes and PEs, split by sex.** In females, A) total number of depressive episodes and B) PEs as outcomes. In males, C) total number of depressive episodes and D) PEs as outcomes. Standardised effect sizes from negative binomial models with 95% CIs displayed as bars. Red asterisks indicate significance using FDR corrected p-values, blue asterisks indicate significance using uncorrected p-values. Level of significance: *: p < 0.05. (For interpretation of the references to colour in this figure legend, the reader is referred to the Web version of this article.)

Results of other associations are described in Supplementary Table 4. Similar effect sizes were observed when missing data was imputed (Supplementary Table 4; Supplementary Fig. 4), when individuals with a self-reported infection (N = 457) were excluded (Supplementary Table 6) and when individuals with CRP ≥ 10 mg/L (N = 60) were included (Supplementary Table 7). Similar effect sizes were also observed between serum IL-6 and total depressive episodes in females and males for both base and fully adjusted models (Supplementary Table 4; Fig. 3).

## 4 Discussion

To our knowledge, this is one of the first studies to investigate early-life inflammation (acute and stable markers) with the subsequent burden of depression and PEs, across early adolescence and into the third decade of life. This follow-up period includes the peak incidence period for major psychiatric disorders and continued neurodevelopment. We found childhood inflammation, as measured by serum IL-6 at age 9 years, was associated with the subsequent burden of depressive episodes and, to a lesser extent, PEs. Further, CRP DNAm scores were weakly related to the burden of PEs later in life (up to 26 years).

Our findings are consistent with previous studies in ALSPAC (Khandaker et al., 2014)^,^(Perry et al., 2021b). For instance, serum IL-6 at age 9 years has been shown to associate with depression and PEs at age 18 years (Khandaker et al., 2014) and depressive episodes, psychotic disorder and negative symptoms at age 24 years (Perry et al., 2021b). Our results extend these findings, by using multiple timepoints of mental health assessments (N = 16) to assess the effect of early-life inflammation on the burden of psychiatric outcomes. Additionally, we utilised novel markers of inflammation that represent a more stable/long-term measure of inflammation. We show that early-life inflammation is associated with the total number of depressive and psychotic experiences from early-adolescence to adulthood. Multiple episodes of depression are an important marker of disorder burden and severity as they are associated with later chronic depression and treatment resistance (Humer et al., 2020; Kendler et al., 2001). Persistent PEs, as opposed to ones that are transient, are also associated with a greater general psychopathology and increased risk for psychotic disorders (Dominguez et al., 2011; Kalman et al., 2019; Rammos et al., 2021). Hence, it is important to investigate what predicts multiple, subsequent occurrences of depression and PEs during an important period of neurodevelopment.

Similar to the above studies in ALSPAC (Khandaker et al., 2014; Perry et al., 2021b), we did not find associations between the acute inflammatory marker serum CRP and later life psychiatric outcomes, potentially due to measurement fluctuations of serum CRP. CRP is also an unspecific marker for acute inflammation and is associated with many different conditions, including non-psychiatric illnesses (Pepys and Hirschfield, 2003). Many previous studies investigating the impact of inflammation on psychiatric outcomes only include acute serum markers of inflammation. However, these markers fluctuate throughout the day and are heavily influenced by recent infection (Bogaty et al., 2013). We therefore investigated novel epigenetic and genetic markers of inflammation, which reflect a longer-term, more stable inflammatory exposure. Other studies, including our own investigating adult population cohorts, have highlighted the importance of researching DNAm markers of inflammation over serum-based markers (Conole et al., 2021; Green et al., 2021; Stevenson et al., 2021). CRP DNAm scores associated with more brain regions and with larger effect sizes than serum CRP, in the context of depression (Green et al., 2021). Further, CRP DNAm scores were also better predictors of cognition than serum measures of CRP (Conole et al., 2021). Here, we found weak associations of CRP DNAm scores with total number of PEs. These associations had larger effect sizes (β) than associations with serum measures but were conducted in smaller samples, which may have contributed to the wide confidence intervals observed and thus lack of significance after correcting for multiple tests. CRP DNAm scores derived from blood at age 7 years showed no evidence for associations in the base model. However, when additionally adjusting for BMI, maternal education and WBC estimates these effect sizes increased, possibly due to multicollinearity. Stronger associations observed for DNAm scores derived from birth rather than age 7 years may be because DNA methylation across the genome is highly sensitive to experiences around the time of birth and this is associated with changes in brain connectivity (Wheater et al., 2022). Additionally, systemic inflammation in the new-born period is strongly associated with atypical brain development (Hagberg et al., 2015; Sullivan et al., 2020). Our findings extend the current literature providing preliminary evidence that DNAm scores of CRP could be important indicators for the burden of mental health outcomes, though further studies in larger samples would be required to confirm this relationship. Further studies should also investigate how early-life DNAm markers of inflammation affect brain development, which may contribute to the development of psychiatric illness.

We observed strong associations between serum IL-6 and total number of depressive episodes, and weaker associations with total number of PEs. Although associations between serum IL-6 and total depressive episodes were not as strong when models were corrected for BMI and maternal education (an indication of socioeconomic status). This is consistent with previous studies showing an effect of BMI and socioeconomic factors in the relationship between inflammation and psychiatric outcomes (Kappelmann et al., 2021a; Muscatell et al., 2020). Despite this potential confounding, MR studies have indicated IL-6 to be potentially causal for schizophrenia and depression (Kappelmann et al., 2021a; Perry et al., 2021a). Additionally, a recent MR study suggests that IL-6 may have causal effects on brain structures relevant to psychiatric disorders (Williams et al., 2022). Therefore, inflammation early in childhood may be associated with depressive episodes and PEs through mediating changes in brain structure and function. Future studies should extend this work further to investigate this. Despite similar effect sizes being observed for both associations of serum IL-6 with total number of depressive episodes and PEs, only the associations with total depressive episodes remained after correcting for multiple testing in the base model (adjusting for sex only). This could be due to differences in power to detect associations with depressive episodes compared to PEs. Depressive episodes were more common than PEs in our sample which would increase power for detecting associations between inflammation and depressive episodes (Supplementary Fig. 2). It is also for this reason that we investigated the total number of PEs across several assessments rather than an outcome of persistent PEs across all time points, despite persistent PEs perhaps being more clinically meaningful (Dominguez et al., 2011). A lack of relationship between inflammatory markers and total number of PEs could also relate to methodological issues, such as the difference in definitions for identifying PEs between studies. Previous ALSPAC studies have shown associations between inflammation and subsequent PEs at single time points at age 18 and 24 years (Khandaker et al., 2014; Perry et al., 2021b). These used an interviewer-determined definition of PEs, based on additional PLIKS-Q questions, available at clinic appointments. Our study maximises data availability by including responses from six remote assessments in addition to the three clinic appointments. For consistency, we only used PLIKS-Q questions available at all time points to define PEs, similar to a previous ALSPAC study (Thapar et al., 2012). We observed similar effect sizes between the main analysis and a sensitivity analysis using the interviewer-rated definition of PEs at the three clinic time points only (Supplementary Fig. 3, Supplementary Table 5). This suggests our findings were not influenced by using different PE definitions. We were unable to perform a similar sensitivity analysis with the depression data in the current study, due to only self-reported data being available. However, we note that the SMFQ is a widely used research tool and we have previously demonstrated the SMFQ has good internal reliability (Kwong, 2019). We also recognise that the SMFQ is not a diagnostic instrument, however scoring ≥ 11 has good specificity for predicting a diagnosis in clinical instruments (Turner et al., 2014) and has been validated in both childhood and adulthood (Eyre et al., 2021; Thapar and McGuffin, 1998). We also note that while prevalence is high, this is highly consistent with previous work suggesting this is likely due to individuals being of the age where important neurodevelopment and onset of psychiatric disorders typically occurs (Thapar et al., 2022).

Despite inflammatory PRSs correlating with their serum equivalents, we did not find associations between inflammatory PRSs and depressive episodes or PEs. This is similar to another study in a large cohort of young individuals (Karcher et al., 2022). This could be due to potential weak power of the PRSs, due to small discovery sample sizes for the GWASs. Additionally, ancestry is important to take into account and is a possible source of bias for PRS analysis. The IL-6 PRS may have been particularly prone to this as the IL-6 GWAS was conduced in Finnish cohorts, which may have different ancestry structure to participants in ALSPAC. Further, PRSs scores were projected from association studies conducted in older individuals. It is known that immune system function changes with age (Montecino-Rodriguez et al., 2013). This may also explain the weaker associations observed with DNAm scores. This difference in ages between the discovery samples and ALSPAC cohort may also have influenced the associations observed between PRSs and DNAm with psychiatric outcomes, as we mostly observed only weak correlations of PRSs and DNAm scores with their serum equivalents. Future studies should also conduct GWASs and epigenome wide association studies (EWASs) in younger cohorts so scores can be calculated from cohorts of similar ages. Finally, using a wider selection of inflammatory markers, rather than being limited to only CRP and IL-6, will also help in understanding which inflammatory pathways are important in the context of psychiatric disorders. Additionally, the lack of associations observed between PRSs and psychiatric outcomes may have been due to the strong effect of environmental factors, such as childhood trauma and chronic physical illnesses, on the immune system. Previous studies have shown relationships of such environmental factors with increased inflammation and psychiatric outcomes (Danese et al., 2008; Flouri et al., 2020).

Potential bias from sample attrition is a possible threat for all longitudinal studies, causing missing data (Biering et al., 2015). Therefore, we imputed missing data for sex, maternal education, BMI (age 7 and 9 years) and depression SMFQ scores and PEs at each time point using multiple imputation (van Buuren and Groothuis-Oudshoorn, 2011). We observed similar effect sizes in the imputed datasets analysis to the complete case main analysis, indicating our results are robust to this potential source of bias.

Our study used the same measures of depression and PEs from 16 assessment points across 18 years, making it one of the most detailed longitudinal studies in a large sample. We were able to go beyond simple acute serum-based markers of inflammation and expand to additionally exploring PRSs and DNAm scores. This enabled us to further our understanding of the effect of inflammation on disorder burden within this important developmental period in adolescence to early adulthood. Our work builds upon the existing evidence showing inflammation in early childhood to be important for psychiatric burden later in life.

## Declaration of competing interest

All authors have nothing to disclose.

## Data Availability

The ALSPAC study website contains details of all data available: http://www.bristol.ac.uk/alspac/researchers/our-data. Analysis code is publicly available: www.github.com/AmeliaES/ALSPAC_inflam _2022

## References

[bib1] Ahola-Olli A.V., Würtz P., Havulinna A.S., Aalto K., Pitkänen N., Lehtimäki T., Kähönen M., Lyytikäinen L.-P., Raitoharju E., Seppälä I., Sarin A.-P., Ripatti S., Palotie A., Perola M., Viikari J.S., Jalkanen S., Maksimow M., Salomaa V., Salmi M., Kettunen J., Raitakari O.T. (2017). Genome-wide association study identifies 27 loci influencing concentrations of circulating cytokines and growth factors. Am. J. Hum. Genet..

[bib2] Barker E.D., Cecil C.A.M., Walton E., Houtepen L.C., O'Connor T.G., Danese A., Jaffee S.R., Jensen S.K.G., Pariante C., Mcardle W., Gaunt T.R., Relton C.L., Roberts S. (2018). Inflammation-related epigenetic risk and child and adolescent mental health: a prospective study from pregnancy to middle adolescence. Dev. Psychopathol..

[bib3] Beydoun M.A., Obhi H.K., Weiss J., Canas J.A., Beydoun H.A., Evans M.K., Zonderman A.B. (2020). Systemic inflammation is associated with depressive symptoms differentially by sex and race: a longitudinal study of urban adults. Mol. Psychiatr..

[bib4] Biering K., Hjollund N.H., Frydenberg M. (2015). Using multiple imputation to deal with missing data and attrition in longitudinal studies with repeated measures of patient-reported outcomes. Clin. Epidemiol..

[bib5] Bogaty P., Dagenais G.R., Joseph L., Boyer L., Leblanc A., Bélisle P., Brophy J.M. (2013). Time variability of C-reactive protein: implications for clinical risk stratification. PLoS One.

[bib6] Boyd A., Golding J., Macleod J., Lawlor D.A., Fraser A., Henderson J., Molloy L., Ness A., Ring S., Davey Smith G. (2013). Cohort profile: the ‘children of the 90s’—the index offspring of the Avon longitudinal study of Parents and children. Int. J. Epidemiol..

[bib7] Chen Y.-A., Lemire M., Choufani S., Butcher D.T., Grafodatskaya D., Zanke B.W., Gallinger S., Hudson T.J., Weksberg R. (2013). Discovery of cross-reactive probes and polymorphic CpGs in the illumina infinium HumanMethylation450 microarray. Epigenetics.

[bib8] Conole E.L.S., Stevenson A.J., Muñoz Maniega S., Harris S.E., Green C., Valdés Hernández M.D.C., Harris M.A., Bastin M.E., Wardlaw J.M., Deary I.J., Miron V.E., Whalley H.C., Marioni R.E., Cox S.R. (2021). DNA methylation and protein markers of chronic inflammation and their associations with brain and cognitive aging. Neurology.

[bib9] Danese A., Moffitt T.E., Pariante C.M., Ambler A., Poulton R., Caspi A. (2008). Elevated inflammation levels in depressed adults with a history of childhood maltreatment. Arch. Gen. Psychiatr..

[bib10] Dominguez M.D.G., Wichers M., Lieb R., Wittchen H.-U., Van Os J. (2011). Evidence that onset of clinical psychosis is an outcome of progressively more persistent subclinical psychotic experiences: an 8-year cohort study. Schizophr. Bull..

[bib11] Eyre O., Bevan Jones R., Agha S.S., Wootton R.E., Thapar A.K., Stergiakouli E., Langley K., Collishaw S., Thapar A., Riglin L. (2021). Validation of the short Mood and Feelings Questionnaire in young adulthood. J. Affect. Disord..

[bib12] Flouri E., Francesconi M., Midouhas E., Lewis G. (2020). Prenatal and childhood adverse life events, inflammation and depressive symptoms across adolescence. J. Affect. Disord..

[bib13] Fraser A., Macdonald-Wallis C., Tilling K., Boyd A., Golding J., Davey Smith G., Henderson J., Macleod J., Molloy L., Ness A., Ring S., Nelson S.M., Lawlor D.A. (2013). Cohort profile: the Avon longitudinal study of Parents and children: ALSPAC mothers cohort. Int. J. Epidemiol..

[bib14] Giollabhui N.M., Ellman L.M., Coe C.L., Byrne M.L., Abramson L.Y., Alloy L.B. (2020). To exclude or not to exclude: considerations and recommendations for C-reactive protein values higher than 10 mg/L. Brain Behav. Immun..

[bib15] Green C., Shen X., Stevenson A.J., Conole E.L.S., Harris M.A., Barbu M.C., Hawkins E.L., Adams M.J., Hillary R.F., Lawrie S.M., Evans K.L., Walker R.M., Morris S.W., Porteous D.J., Wardlaw J.M., Steele J.D., Waiter G.D., Sandu A.-L., Campbell A., Marioni R.E., Cox S.R., Cavanagh J., Mcintosh A.M., Whalley H.C. (2021). Structural brain correlates of serum and epigenetic markers of inflammation in major depressive disorder. Brain Behav. Immun..

[bib16] Hagberg H., Mallard C., Ferriero D.M., Vannucci S.J., Levison S.W., Vexler Z.S., Gressens P. (2015). The role of inflammation in perinatal brain injury. Nat. Rev. Neurol..

[bib17] Haroon E., Daguanno A.W., Woolwine B.J., Goldsmith D.R., Baer W.M., Wommack E.C., Felger J.C., Miller A.H. (2018). Antidepressant treatment resistance is associated with increased inflammatory markers in patients with major depressive disorder. Psychoneuroendocrinology.

[bib18] Houseman E.A., Accomando W.P., Koestler D.C., Christensen B.C., Marsit C.J., Nelson H.H., Wiencke J.K., Kelsey K.T. (2012). DNA methylation arrays as surrogate measures of cell mixture distribution. BMC Bioinf..

[bib19] Humer E., Kocsis-Bogar K., Berger T., Schröder J., Späth C., Meyer B., Moritz S., Lutz W., Probst T., Klein J.P. (2020). A comparison of the three year course between chronic depression and depression with multiple vs. few prior episodes. Psychiatr. Res..

[bib20] Iob E., Kirschbaum C., Steptoe A. (2020). Persistent depressive symptoms, HPA-axis hyperactivity, and inflammation: the role of cognitive-affective and somatic symptoms. Mol. Psychiatr..

[bib21] Kalman J.L., Bresnahan M., Schulze T.G., Susser E. (2019). Predictors of persisting psychotic like experiences in children and adolescents: a scoping review. Schizophr. Res..

[bib22] Kappelmann N., Arloth J., Georgakis M.K., Czamara D., Rost N., Ligthart S., Khandaker G.M., Binder E.B. (2021). Dissecting the association between inflammation, metabolic dysregulation, and specific depressive symptoms. JAMA Psychiatr..

[bib23] Kappelmann N., Czamara D., Rost N., Moser S., Schmoll V., Trastulla L., Stochl J., Lucae S., Binder E.B., Khandaker G.M., Arloth J. (2021). Polygenic risk for immuno-metabolic markers and specific depressive symptoms: a multi-sample network analysis study. Brain Behav. Immun..

[bib24] Karcher N.R., Paul S.E., Johnson E.C., Hatoum A.S., Baranger D.A.A., Agrawal A., Thompson W.K., Barch D.M., Bogdan R. (2022). Psychotic-like experiences and polygenic liability in the adolescent brain cognitive development study. Biol. Psychiatr.: Cognitive Neuroscience and Neuroimaging.

[bib25] Kendler K.S., Thornton L.M., Gardner C.O. (2001). Genetic risk, number of previous depressive episodes, and stressful life events in predicting onset of major depression. Am. J. Psychiatr..

[bib26] Khandaker G.M., Pearson R.M., Zammit S., Lewis G., Jones P.B. (2014). Association of serum interleukin 6 and C-reactive protein in childhood with depression and psychosis in young adult life. JAMA Psychiatr..

[bib27] Khandaker G.M., Stochl J., Zammit S., Goodyer I., Lewis G., Jones P.B. (2018). Childhood inflammatory markers and intelligence as predictors of subsequent persistent depressive symptoms: a longitudinal cohort study. Psychol. Med..

[bib28] Kwong A.S.F. (2019). Examining the longitudinal nature of depressive symptoms in the Avon longitudinal study of Parents and children (ALSPAC). Wellcome Open Research.

[bib29] Kwong A.S.F., López-López J.A., Hammerton G., Manley D., Timpson N.J., Leckie G., Pearson R.M. (2019). Genetic and environmental risk factors associated with trajectories of depression symptoms from adolescence to young adulthood. JAMA Netw. Open.

[bib30] Kwong A.S.F., Morris T.T., Pearson R.M., Timpson N.J., Rice F., Stergiakouli E., Tilling K. (2021). Polygenic risk for depression, anxiety and neuroticism are associated with the severity and rate of change in depressive symptoms across adolescence. JCPP (J. Child Psychol. Psychiatry).

[bib31] Ligthart S., Marzi C., Aslibekyan S., Mendelson M.M., Conneely K.N., Tanaka T., Colicino E., Waite L.L., Joehanes R., Guan W., Brody J.A., Elks C., Marioni R., Jhun M.A., Agha G., Bressler J., Ward-Caviness C.K., Chen B.H., Huan T., Bakulski K., Salfati E.L., Fiorito G., Wahl S., Schramm K., Sha J., Hernandez D.G., Just A.C., Smith J.A., Sotoodehnia N., Pilling L.C., Pankow J.S., Tsao P.S., Liu C., Zhao W., Guarrera S., Michopoulos V.J., Smith A.K., Peters M.J., Melzer D., Vokonas P., Fornage M., Prokisch H., Bis J.C., Chu A.Y., Herder C., Grallert H., Yao C., Shah S., Mcrae A.F., Lin H., Horvath S., Fallin D., Hofman A., Wareham N.J., Wiggins K.L., Feinberg A.P., Starr J.M., Visscher P.M., Murabito J.M., Kardia S.L.R., Absher D.M., Binder E.B., Singleton A.B., Bandinelli S., Peters A., Waldenberger M., Matullo G., Schwartz J.D., Demerath E.W., Uitterlinden A.G., Van Meurs J.B.J., Franco O.H., Chen Y.-D.I., Levy D., Turner S.T., Deary I.J., Ressler K.J., Dupuis J., Ferrucci L., Ong K.K., Assimes T.L., Boerwinkle E., Koenig W., Arnett D.K., Baccarelli A.A., Benjamin E.J., Dehghan A. (2016). DNA methylation signatures of chronic low-grade inflammation are associated with complex diseases. Genome Biol..

[bib32] Lloyd-Jones L.R., Zeng J., Sidorenko J., Yengo L., Moser G., Kemper K.E., Wang H., Zheng Z., Magi R., Esko T., Metspalu A., Wray N.R., Goddard M.E., Yang J., Visscher P.M. (2019). Improved polygenic prediction by Bayesian multiple regression on summary statistics. Nat. Commun..

[bib33] Metcalf S.A., Jones P.B., Nordstrom T., Timonen M., Mäki P., Miettunen J., Jääskeläinen E., Järvelin M.-R., Stochl J., Murray G.K., Veijola J., Khandaker G.M. (2017). Serum C-reactive protein in adolescence and risk of schizophrenia in adulthood: a prospective birth cohort study. Brain Behav. Immun..

[bib34] Montecino-Rodriguez E., Berent-Maoz B., Dorshkind K. (2013). Causes, consequences, and reversal of immune system aging. J. Clin. Invest..

[bib35] Muscatell K.A., Brosso S.N., Humphreys K.L. (2020). Socioeconomic status and inflammation: a meta-analysis. Mol. Psychiatr..

[bib36] Northstone K., Lewcock M., Groom A., Boyd A., Macleod J., Timpson N., Wells N. (2019). The Avon Longitudinal Study of Parents and Children (ALSPAC): an update on the enrolled sample of index children in 2019. Wellcome Open Res.

[bib37] Osimo E.F., Stochl J., Zammit S., Lewis G., Jones P.B., Khandaker G.M. (2020). Longitudinal population subgroups of CRP and risk of depression in the ALSPAC birth cohort. Compr. Psychiatr..

[bib38] Pepys M.B., Hirschfield G.M. (2003). C-reactive protein: a critical update. J. Clin. Invest..

[bib39] Perry B.I., Upthegrove R., Kappelmann N., Jones P.B., Burgess S., Khandaker G.M. (2021). Associations of immunological proteins/traits with schizophrenia, major depression and bipolar disorder: a bi-directional two-sample mendelian randomization study. Brain Behav. Immun..

[bib40] Perry B.I., Zammit S., Jones P.B., Khandaker G.M. (2021). Childhood inflammatory markers and risks for psychosis and depression at age 24: examination of temporality and specificity of association in a population-based prospective birth cohort. Schizophr. Res..

[bib41] Purcell S., Neale B., Todd-Brown K., Thomas L., Ferreira M.A.R., Bender D., Maller J., Sklar P., De Bakker P.I.W., Daly M.J., Sham P.C. (2007). PLINK: a tool set for whole-genome association and population-based linkage analyses. Am. J. Hum. Genet..

[bib42] Rammos A., Sullivan S.A., Kounali D., Jones H.J., Hammerton G., Hines L.A., Lewis G., Jones P.B., Cannon M., Thompson A., Wolke D., Heron J., Zammit S. (2021). Precursors and correlates of transient and persistent longitudinal profiles of psychotic experiences from late childhood through early adulthood. Br. J. Psychiatr..

[bib43] Relton C.L., Gaunt T., Mcardle W., Ho K., Duggirala A., Shihab H., Woodward G., Lyttleton O., Evans D.M., Reik W., Paul Y.-L., Ficz G., Ozanne S.E., Wipat A., Flanagan K., Lister A., Heijmans B.T., Ring S.M., Davey Smith G. (2015). Data resource profile: accessible resource for integrated epigenomic studies (ARIES). Int. J. Epidemiol..

[bib44] Sproston N.R., Ashworth J.J. (2018). Role of C-reactive protein at sites of inflammation and infection. Front. Immunol..

[bib45] Stevenson A.J., Gadd D.A., Hillary R.F., Mccartney D.L., Campbell A., Walker R.M., Evans K.L., Harris S.E., Spires-Jones T.L., Mcrae A.F., Visscher P.M., Mcintosh A.M., Deary I.J., Marioni R.E. (2021).

[bib46] Sullivan G., Galdi P., Cabez M.B., Borbye-Lorenzen N., Stoye D.Q., Lamb G.J., Evans M.J., Quigley A.J., Thrippleton M.J., Skogstrand K., Chandran S., Bastin M.E., Boardman J.P. (2020). Interleukin-8 dysregulation is implicated in brain dysmaturation following preterm birth. Brain Behav. Immun..

[bib47] Thapar A., Eyre O., Patel V., Brent D. (2022). Depression in young people. Lancet.

[bib48] Thapar A., Heron J., Jones R.B., Owen M.J., Lewis G., Zammit S. (2012). Trajectories of change in self-reported psychotic-like experiences in childhood and adolescence. Schizophr. Res..

[bib49] Thapar A., McGuffin P. (1998). Validity of the shortened Mood and Feelings Questionnaire in a community sample of children and adolescents: a preliminary research note. Psychiatr. Res..

[bib50] Turner N., Joinson C., Peters T.J., Wiles N., Lewis G. (2014). Validity of the short Mood and Feelings Questionnaire in late adolescence. Psychol. Assess..

[bib51] van Buuren S., Groothuis-Oudshoorn K. (2011). Mice: multivariate imputation by chained Equations in R. J. Stat. Software.

[bib52] Visser M. (1999). Elevated C-reactive protein levels in overweight and obese adults. JAMA.

[bib53] Wheater E.N.W., Galdi P., Mccartney D.L., Blesa M., Sullivan G., Stoye D.Q., Lamb G., Sparrow S., Murphy L., Wrobel N., Quigley A.J., Semple S., Thrippleton M.J., Wardlaw J.M., Bastin M.E., Marioni R.E., Cox S.R., Boardman J.P. (2022). DNA methylation in relation to gestational age and brain dysmaturation in preterm infants. Brain Communications.

[bib54] Williams J.A., Burgess S., Suckling J., Lalousis P.A., Batool F., Griffiths S.L., Palmer E., Karwath A., Barsky A., Gkoutos G.V., Wood S., Barnes N.M., David A.S., Donohoe G., Neill J.C., Deakin B., Khandaker G.M., Upthegrove R., Rogers J.C., Mondelli V., Dazzan P., Pariante C., Maccabe J., Egerton A., Jones P., Bullmore E., Koutsouleris N., Meisenzahl E., Cotter D., Harrison N. (2022). Inflammation and brain structure in schizophrenia and other neuropsychiatric disorders. JAMA Psychiatr..

[bib55] Yuan N., Chen Y., Xia Y., Dai J., Liu C. (2019). Inflammation-related biomarkers in major psychiatric disorders: a cross-disorder assessment of reproducibility and specificity in 43 meta-analyses. Transl. Psychiatry.

